# Effective Mean Square Differences: A Matching Algorithm for Highly Similar Sheet Metal Parts

**DOI:** 10.3390/s23167300

**Published:** 2023-08-21

**Authors:** Hui Zhang, Zhen Guan, Joe Eastwood, Hongji Zhang, Xiaoyang Zhu

**Affiliations:** 1 School of Mechanical Engineering, Jiangsu University of Science and Technology, Zhenjiang 212100, China; 2 Manufacturing Metrology Team, Faculty of Engineering, University of Nottingham, Nottingham NG8 1BB, UK

**Keywords:** sheet metal parts identification, highly similar parts, matching algorithm, image pyramid

## Abstract

The accurate identification of highly similar sheet metal parts remains a challenging issue in sheet metal production. To solve this problem, this paper proposes an effective mean square differences (EMSD) algorithm that can effectively distinguish highly similar parts with high accuracy. First, multi-level downsampling and rotation searching are adopted to construct an image pyramid. Then, non-maximum suppression is utilised to determine the optimal rotation for each layer. In the matching, by re-evaluating the contribution of the difference between the corresponding pixels, the matching weight is determined according to the correlation between the grey value information of the matching pixels, and then the effective matching coefficient is determined. Finally, the proposed effective matching coefficient is adopted to obtain the final matching result. The results illustrate that this algorithm exhibits a strong discriminative ability for highly similar parts, with an accuracy of 97.1%, which is 11.5% higher than that of the traditional methods. It has excellent potential for application and can significantly improve sheet metal production efficiency.

## 1. Introduction

Sheet metal parts are widely used in aerospace, household appliances, automobiles, and other fields due to their advantages of a high specific strength, excellent electromagnetic shielding ability, low production cost, and easy mass production [1,2,3,4,5,6,7]. Similarly, image-matching technology is widely used in modern intelligent manufacturing as it can realise automatic production processes and quality control and provide a more efficient, accurate, and reliable solution for the production of sheet metal parts [8,9,10,11]. Sheet metal parts are usually produced in multiple varieties and small batches [12], and are usually gathered together for spray painting and other surface treatment processes [13]. After spray painting, various sheet metal parts must be identified again [14]. Completing this recognition task quickly and accurately is important for improving the quality and efficiency of sheet metal parts production.

With the continuous improvement and innovation in computer vision and machine learning technology, image recognition has been extensively adopted in industry. Cusano et al. used template matching and local operator matching methods to locate and classify eight types of operation panels and 20 different parts in an Alenia-Aermacchi M346 aircraft under maintenance, and the classification accuracy reached 83.7% [15]. Machine-learning methods such as convolutional neural networks (CNNs) have also become widely adopted. For instance, in the context of part classification, Guo Fei et al. proposed an enhanced part recognition algorithm that can successfully identify multiple parts of the same type amidst complex backgrounds, achieving an impressive recognition accuracy rate of 98.8% for screws and nuts [16]. Hou et al. [17] introduced a positioning technology using a template-matching algorithm for high-precision positioning that can adapt to different target changes and motion trajectories and has a high positioning accuracy. Although satisfactory results have been achieved, sheet metal parts, such as those investigated in this study, are often highly similar, and the feature difference area is very small compared with the overall image of the sheet metal parts. This high similarity makes it extremely difficult to accurately and efficiently perform recognition tasks on sheet metal parts.

To solve the problem of image recognition in production, template-matching methods and deep learning methods are often used based on greyscale pixel values. However, to identify images with a high similarity, a deeper and more complex network structure and substantial amounts of labelling data are required. Meanwhile, the production of sheet metal parts usually adopts the routines of multiple varieties and small batches, which makes it difficult to organise highly automated production lines and relies on high-intensity manual labour, making it quite difficult to collect large amounts of datasets for label information [18,19,20]. Therefore, machine-learning techniques are not suitable for this application. As image matching depends on grey information, the sum of squared differences (SSD) [21] and sum of absolute differences (SAD) [21] algorithms use different error measurement methods. Both algorithms involve calculating the difference between the corresponding pixel values of the target and template images and identifying the template by comparing the corresponding pixel values of each area [22]. Although these two methods are easy to implement, the matching speed is slow and sensitive to changes in lighting conditions. Various algorithms have been proposed to improve the anti-interference ability and matching speed of the algorithms. The normalised cross-correlation (NCC) [23] algorithm uses a normalised correlation coefficient to evaluate the grey-matching level between matched images. Compared with the SSD and SAD algorithms, the NCC algorithm shows stronger robustness and can cope better with noise and illumination changes. However, the NCC algorithm needs to traverse all pixels in the image and has a complex similarity measurement formula, resulting in a large number of calculations and a slow matching speed. A rotation-invariant image-matching algorithm has been proposed [24], whose matching speed is faster than that of NCC. This algorithm uses an effective rotation-invariant measurement method that can accurately match the template by rotation and it achieves a high matching accuracy. Although this method guarantees a matching accuracy, it has a few limitations in terms of the accurate matching of the differential features of similar objects, and its matching speed has significant room for improvement. Regarding engineering applications, Li et al. [25] analysed various changing factors in the sheet metal forming process and extracted specific surface features for the optimal design of the process layout.

This study proposes an efficient mean square deviation (EMSD) algorithm for identifying highly similar parts based on greyscale image information. First, multi-level downsampling and rotation searching are adopted to construct an image pyramid. Then, non-maximum suppression is utilised to determine the optimal rotation at each layer. Next, we calculate the image matching effectiveness between the images to be recognised in order to better describe the differences between highly similar parts and achieve accurate recognition of similar parts.

The remainder of this paper is organised as follows: Section 2 describes the distinctive features of high-similarity sheet metal parts. Section 3 describes the EMSD image-matching algorithm. Experimental procedures are presented in Section 4. Section 5 summarises the conclusions of the study.

## 2. Characteristics of Highly Similar Sheet Metal Parts

**Figure 1:**
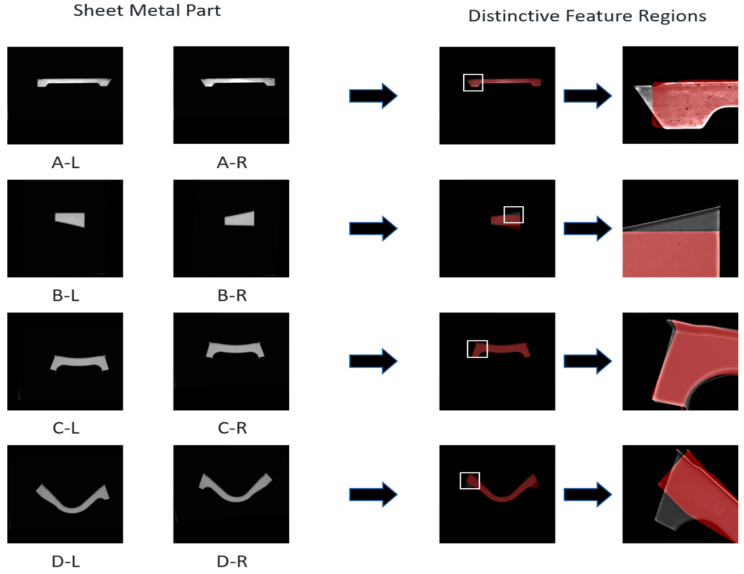
Image matching of similar sheet metal parts.

Before explaining the principle of the algorithm, it is necessary to analyse the different characteristics of sheet metal parts with a high similarity. In the image matching process, it is difficult to accurately match symmetric metal parts with only slight differences in a local curved-edge curvature. These differences are considerably small compared with the overall size of the parts. Figure 1 illustrates four pairs of highly similar sheet metal parts, with the mask displaying a differentiated feature region of the sheet metal part. Distinctive feature regions are shown as red masks within the magnified view.

**Figure 2:**
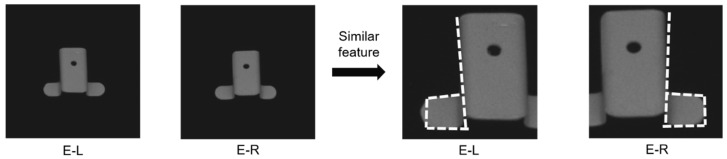
Similar features of similar sheet metal parts.

Figure 2 shows a pair of highly similar metal parts with two sets of curved features, one of which has different curvature angles in the vertical direction. It is difficult to directly distinguish between the two sheet metal parts by observing the original image of this pair of sheet metal parts. Furthermore, to demonstrate the differences in small areas, the differentiated feature regions of this pair of sheet metal parts are marked with guidelines. These differentiated features are highly similar and symmetrical. The precise matching of this type of sheet metal parts poses a more challenging task.

## 3. Feature Analysis of Highly Similar Sheet Metal Parts

First, multi-level downsampling and rotation searching are adopted to construct an image pyramid for acceleration [26,27,28,29]. In the matching process for each layer, the target image and template image are rotated several times by a certain angle. The calculation of the next layer will start from the optimal rotation angle of the upper layer, and then the target image is rotated multiple times at a smaller angle for a precise matching calculation. The rotation angle is obtained layer by layer. This step is explained in detail in Section 3.1. Second, the effective matching coefficient is adopted to calculate the difference in information between the images to be matched. As the parts’ images in this study are grey and the background is black, the effective matching coefficient directly indicates differences according to the grey level of the pixels, so as to better describe the differences between images. This step is explained in detail in Section 3.2. Finally, the EMSD algorithm uses the effective matching coefficient to improve the mean square error matching in order to achieve higher precision matching results.

### 3.1. Rotating Target Search Method Based on the Image Pyramid

By constructing an image pyramid for each template image, the search speed of the target image can be effectively improved compared with that of the ergodic method. Furthermore, it is reasonably applicable to rotated and other disturbing images.

**Figure 3:**
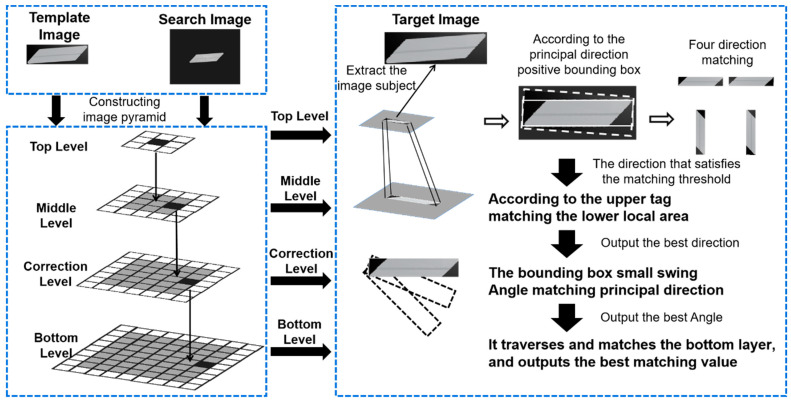
Rotating target search method.

As shown in Figure 3, the search method operates as follows. First, all the original images are built into a series of images as image pyramids with different scales to improve efficiency. Then, at the top level, the search images are traversed with a template image to find the potential target image. All of the matching results are screened using the non-maximum suppression method [30]. Third, the current matching layer starts to search according to the angle and position information passed from the upper layer matching result. The rotation step angle is set to 1 degree in the first layer, and the rotation step angle in the subsequent layer is gradually reduced by an order of magnitude. Finally, the target positioning and rotation angles are obtained. With the image pyramid, the speed and accuracy can be effectively improved.

**Figure 4:**
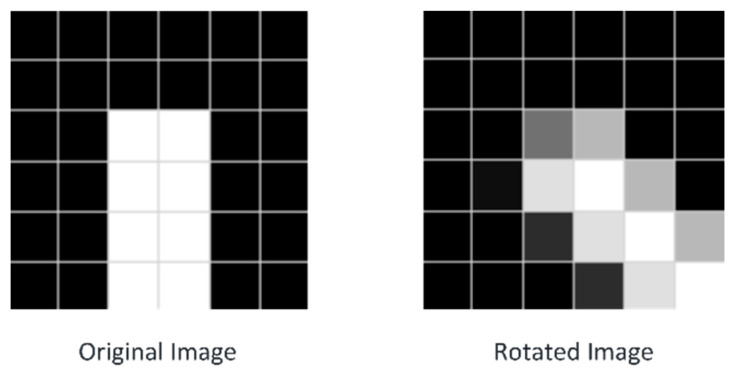
Rotated rectangle target images.

Both image downsampling and rotation create jagged edges in the image contour, as shown in Figure 4. Therefore, in the process of image downsampling, retaining the image details as much as possible while reducing the image resolution is challenging [31]. To solve this problem, an interpolation method is required to fill the pixel gap and reduce its impact on the image edge information. Meanwhile, the application discussed in this study does not need to reproduce the high-precision contour of the original image; thus, a method to minimise the impact caused by the sawtooth edge destroying the contour information is adopted. The proposed algorithm adopts the bilinear interpolation method for image downsampling because it can better retain image contour information [32,33,34]. When downsampling the contour edge of the target, this method can make the contour edge smoother so that the algorithm can adapt to the interference of different rotation angles of the image.

In summary, the hierarchical search algorithm used in this study uses bilinear interpolation to construct the image pyramid and adds rotational image pairing to the search process. In the experiments, a good matching accuracy can be achieved by rotating the image at different levels using different step sizes.

### 3.2. Optimising Image Matching by Effective Matching Coefficient

**Figure 5:**
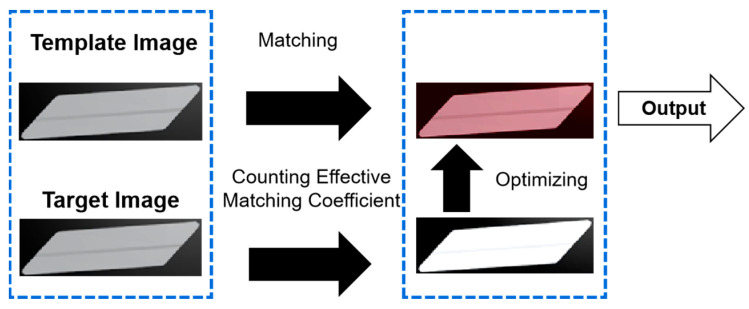
Optimizing the matching process.

The template image is matched to the target image based on the target image identified in the previous step. Subsequently, the matching coefficient is obtained. This process is shown in Figure 5.

Image matching is a basic technology in the fields of image processing and computer vision. This study aimed to address current problems in the production of sheet metal parts by using image-matching technology. In such an application, after determining the posture of the camera and other related hardware and sheet metal parts, the optimal visual system state can be set according to the relationship between the camera and the target [35]. Subsequently, as the layout of the visual system would remain unchanged, the target can be matched using known images. In this case, the mean squared difference (MSD) algorithm is selected in this study for image matching. The similarity measurement formula for this process is as follows [27,28]:

(1) $$D\left(i,j\right)=\frac{1}{m\times n}\sum_{a=1}^{m}\sum_{b=1}^{n}{\left[S(i+a,j+b)-T(a,b)\right]}^{2}$$

(2) $$\left\{\begin{array}{c} 1\leq i\leq M-m+1\\ 1\leq j\leq N-n+1 \end{array}\right.$$

where S(x,y) and $T\left(x,y\right)$ represent the pixel grey values at the corresponding positions of the search and template images, respectively. M, N represents the size of the template image. m, n represents the size of the search image. Obviously, the smaller the mean square variance, the more similar the images. Therefore, it is necessary to find the minimum D(i,j) in order to determine the best location of the matching sub-image. The MSD algorithm performs very well in the recognition of similar targets, and its advantages are mainly reflected in the following aspects. First, the algorithm can effectively resist noise and environmental changes and has good adaptability, as it can adapt to targets of different scales. Second, as the MSD algorithm has a low computing cost and few computing resource requirements, and the image-matching task can be completed in a short time.

**Figure 6:**
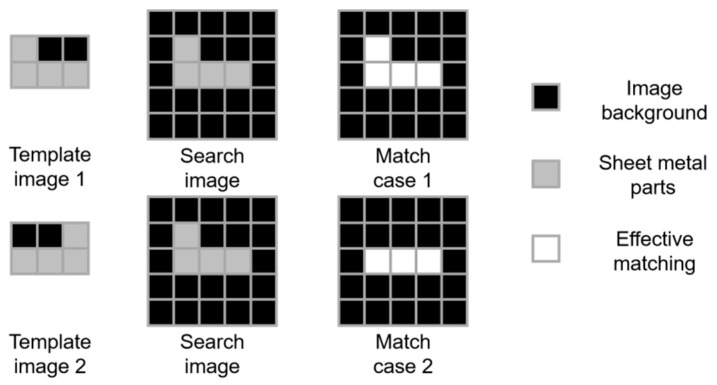
Calculation principle of the effective matching coefficient.

Although the MSD algorithm has practical usefulness and technical advantages, it still has a high error rate for the recognition of highly similar sheet metal parts. In this study, the effective matching coefficient is proposed to optimise image matching. As shown in Figure 6, the three areas are defined as follows: image background, sheet metal parts, and effective matching. The difference operation of a corresponding pixel in the image-matching process is defined as pixel matching. First, each matching pixel is classified based on the location of the pixel area. Meanwhile, the matching weight is not a fixed value, but a normalised value of the corresponding grey value of each pixel. When the matching position is within the part area, the pixel grey value is closer to 255, and the corresponding pixel matching weight is higher. By counting the results of all the valid matches, the matching effectiveness of Case 1 is evidently better than that of Case 2.

In addition, when matching a pair of high-similarity sheet metal part images, different weights are assigned to the corresponding positions of the pixel matches during the matching process. The weight size is measured as the difference between the greyscale values corresponding to the pixel-matching position in the template image and the matching-point pixel. For example, when the pixel-matching positions are located in both the background and sheet metal areas of the image, and this matching position corresponds to the sheet metal area in the template image, this match is a high-weight match. Finally, the calculated results for all pixels are weighted and normalised to obtain an effective matching coefficient. This method enhances the effectiveness of contour difference information and improves the accuracy and robustness of image matching. The similarity estimation equation after image-matching optimisation is as follows:

(3) $$F\left(i,j\right)=\frac{E(i,j)}{m\times n}\sum_{a=1}^{m}\sum_{b=1}^{n}{\left[S(i+a,j+b)-T(a,b)\right]}^{2}$$

Here, E represents the effective matching coefficient, which can be calculated by the following equation:

(4) $$E(i,j)=\frac{\sum_{a=1}^{m}\sum_{b=1}^{n}T(a,b)-\sum_{a=1}^{m}\sum_{b=1}^{n}\sqrt{\left(k-\left|S(i+a,j+b)-T(a,b)\right|\right)\times T(a,b)}}{\sum_{a=1}^{m}\sum_{b=1}^{n}T(a,b)}$$

Here, coefficient k is the maximum pixel grey value of the image. Additionally, the smaller the mean absolute difference, F(i,j), the higher the similarity; therefore, to find the smallest F(i,j), only the matching locations need to be determined. Based on the greyscale difference between the template and search image, the overall similarity estimate values should range from zero to one.

## 4. Experiment

In the experiment, five pairs of sheet metal parts with a high similarity, namely 10 different types of sheet metal parts for recognition, were tested using the proposed method. The images of these sheet metal parts were obtained with different angles using an experimental platform. Then, the matching accuracy and speed of the EMSD algorithm were evaluated under various conditions such as blur, noise, and rotation. The matching accuracy is defined as the ratio of accurately matched images to the total number of images, while the matching speed is the average matching time for all of the images.

**Figure 7:**
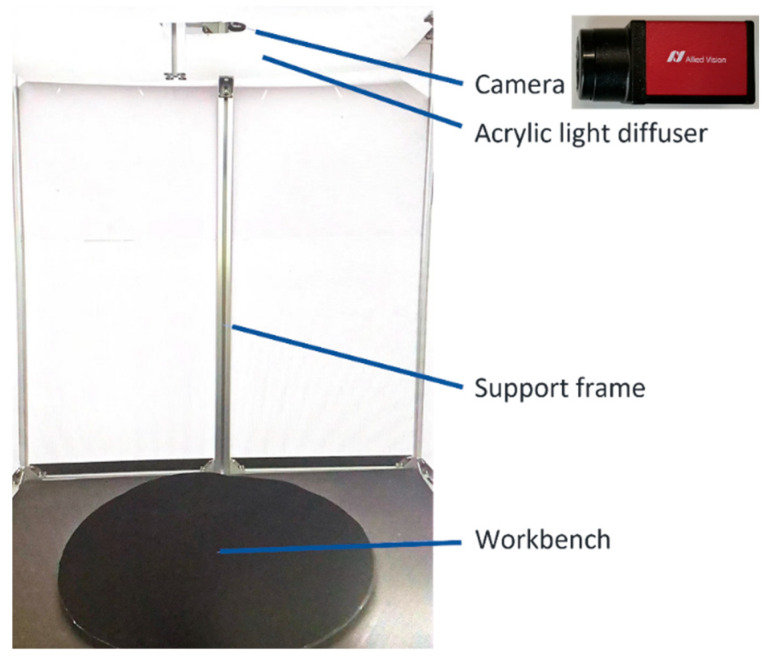
Experiment layout.

The experimental platform consisted of a camera, an acrylic levelling plate, a supporting structure, and a workbench, as shown in Figure 7. An AVT Mako G-158 camera was used for the image acquisition. The algorithm was implemented using C++ on a 64-bit Windows 10 operating system on an Intel Core i5-8400 CPU with 32 GB of memory.

**Figure 8:**
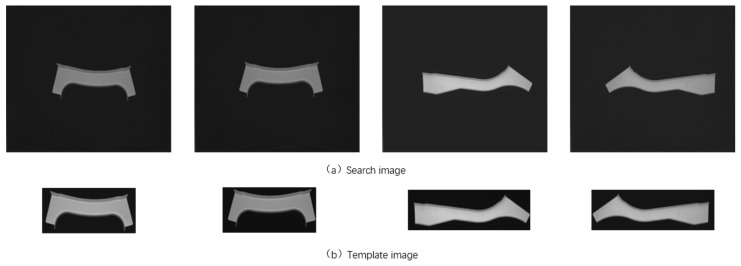
Part of the test image.

**Figure 9:**
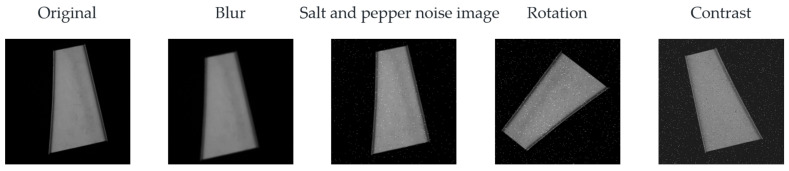
Image noise and interference.

The search images in the experiment were images of parts with high a similarity that were difficult to distinguish in the actual sheet metal production process, and the resolution of the images was 1456 × 1088. The template image in the experiment was extracted from the search image, and the resolution of the image was 550 × 550. The overall shapes of the sheet metal parts in the search image were similar, with symmetric local features in the horizontal and vertical directions in some areas. Figure 8 shows the search and template images. During production, sheet metal part images may encounter interferences, as shown in Figure 9, including noise, blur, rotation, and change contrast. The experiment was conducted in two stages. The first was to test the algorithm using simulation data, and the second was to conduct identification using the real part image.

### 4.1. Algorithm Verification Using Simulation Data

Interferences such as salt-and-pepper noise, Gaussian blur, rotation, and change contrast were added to the corresponding search images, and, finally, 9720 test images were obtained. The image data collection method is shown in Table 1. The test images were input into a recognition test program [36].

### 4.2. Sheet Metal Part Recognition Experiment

**Figure 10:**
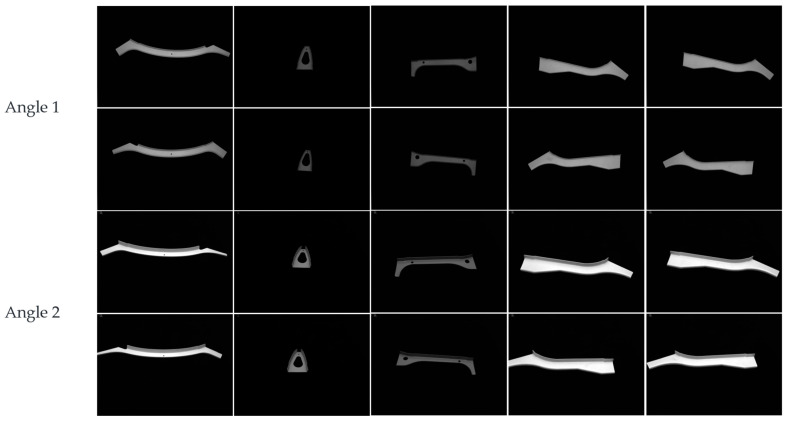
Part of the sheet metal image.

All of the test data were collected from the test platform, as shown in Figure 7. A total of 200 sheet metal parts of different types were selected, as shown in Figure 10. All of the sheet metal parts were placed on the workbench individually for shooting, and the sheet metal images captured from multiple angles corresponded to their serial numbers individually and were stored in the database. Subsequently, the sheet metals recorded in the database were identified. Different sheet metal parts were individually placed on the workbench, photographed, and identified, and the code of the sheet metal part was finally obtained.

## 5. Results

The template image corresponding to each sheet metal component was used to classify each test drawing. The images were divided into five categories according to the interference type: original, salt-and-pepper noise, Gaussian blur, rotation, and contrast change. This study evaluated the performance of the algorithm by testing its matching accuracy and speed and compared it with the original NCC, improved NCC, improved sequential similarity detection algorithm (SSDA), and VGG-16 and MSD algorithms [27,34,35].

### 5.1. Comparison of Matching Speed

**Figure 11:**
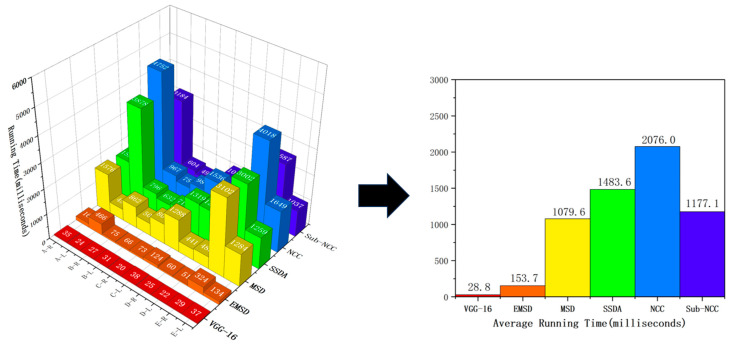
Comparison of implementations of various algorithms.

Figure 11 shows the corresponding running time data for the aforementioned algorithms when processing the 10 selected parts. According to the experimental data, we identified the differences in the running time required by different algorithms when processing images of different parts. Compared with other algorithms, the EMSD algorithm can significantly reduce the processing time. For example, for parts A-R, the NCC algorithm had an average runtime of 2076 ms, while the EMSD algorithm only required 169 ms to complete the same task. The same trend was observed for other parts. All of the algorithms used the same method for coarse screening from multiple parts with matches, and then the same process was used for image matching based on this screening. The NCC and sub-NCC algorithms are both based on cross-correlation methods used to calculate the similarity between two images. In the process of target image location, the EMSD algorithm can significantly shorten the time of image matching by searching layer by layer in the image pyramid, compared with the violent traversal process on the original resolution image. Similarly, improving the accuracy of the rotation angle matching layer by layer in the rotating image and searching only in the upper adjacent range of rotation angles could positively contribute to enhancing the matching speed. Based on the matching average time of the test in this study, the proposed EMSD algorithm is prioritised in terms of time efficiency.

### 5.2. Impact of the Effectiveness Coefficient

**Figure 12:**
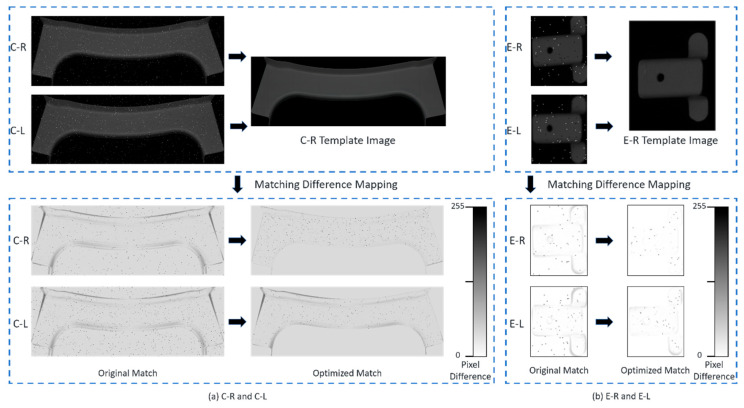
Sheet metal part pixel difference mapping.

The effective match score was obtained using a proportional coefficient formula that calculates the symmetric feature area ratio; the smaller the match score, the higher the degree of matching similarity. When matching sheet metal parts with a high similarity in actual images, interference such as blur and noise may cause the matching scores of the original- and symmetrical-part images to be very close. The C-R image of the sheet metal parts was taken as the image to be classified for matching with the C-R and C-L images of the sheet metal parts. Figure 12 shows a mapping image of the pixel difference values in image matching. After optimising the matching process, it was easier to distinguish the different results of the matched images compared with the results before optimisation. When the relative scale of the symmetric feature size of two similar symmetric sheet metal parts was small compared with the overall scale and the symmetric features were not obvious, the matching score of the original part might be greater than that of the symmetric part, resulting in incorrect matching results. Table 2 displays the effective match scores corresponding to each part, after analysing the experimental data for the 10 parts.

Strengthening the correlation between symmetric feature regions in image matching through effective match scores and correcting the matching results can achieve a good recognition effect for similar symmetric sheet metal parts.

Table 3 shows that the matching scores of the original and symmetric parts are very close, with most of the matching scores for the symmetric parts being smaller than those of the original parts. After effective match score correction, the correct image-matching results were obtained.

### 5.3. Analysis of Algorithm Recognition Accuracy

In the task of handling similar and symmetric targets, according to the experimental results, the EMSD algorithm performed better than the other template-matching algorithms, with a higher matching accuracy and speed. Compared with the MSD algorithm, the EMSD algorithm significantly improved the anti-interference ability and matching speed. The performances of the improved NCC and NCC algorithms were relatively poor when processing noisy and blurred images, with a relatively low accuracy. The MAD algorithm performance was average when processing noisy and blurred images; however, it performed well when dealing with rotated images. As the optimal recognition algorithm, the EMSD algorithm has the advantage of better robustness and recognition ability and can quickly match image features. Therefore, this algorithm can be used in actual symmetric target recognition tasks to achieve a higher recognition rate and shorter processing time.

In the task of handling similar and symmetric targets, according to the results in Table 4, the EMSD algorithm performed better than the other template-matching algorithms, with a higher matching accuracy and speed. Compared with the MSD algorithm, the EMSD algorithm significantly improved the anti-interference ability and matching speed. The improved NCC and NCC algorithms performed relatively poorly when processing noisy and blurred images, with a lower accuracy. The MAD algorithm performed moderately when processing noisy and blurred images, but performed well when dealing with rotated images. The EMSD algorithm has the advantage of better robustness and recognition ability and can quickly match the image features. Therefore, this algorithm can be used in actual symmetric target recognition tasks to achieve a higher recognition rate with a shorter processing time.

### 5.4. Misidentification Factors in Sheet Metal Recognition

**Figure 13:**
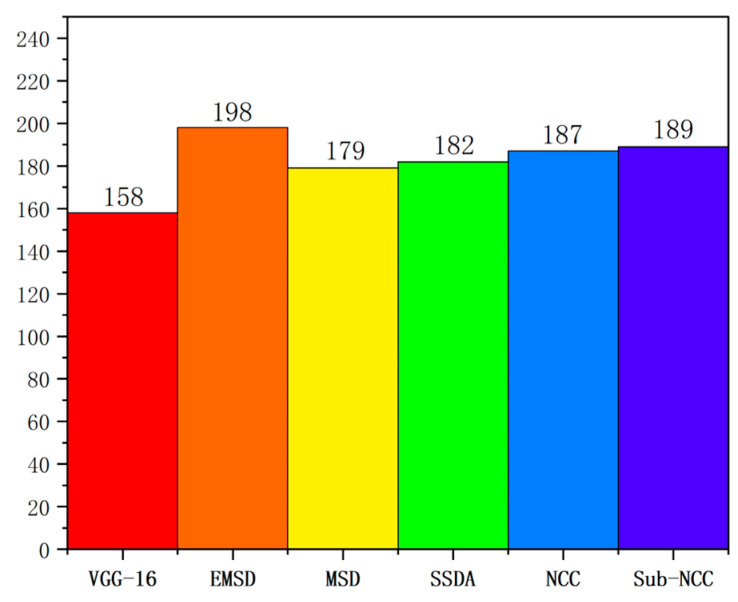
Total of properly detected objects.

Figure 13 shows the results obtained using each of the investigated algorithms in terms of the recognition of 200 types of sheet metal parts. The EMSD algorithm showed the highest recognition accuracy (99%). However, two types of sheet metal parts were misidentified by the proposed EMSD algorithm as well as other matching algorithms.

**Figure 14:**
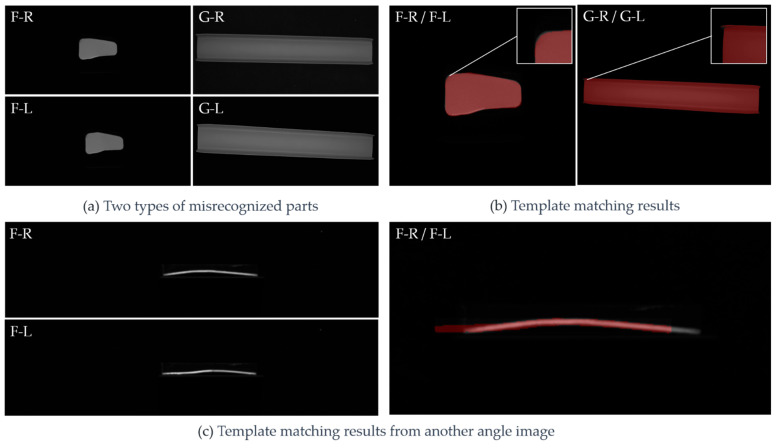
Misidentified cases with further analysis.

Two parts, as shown in Figure 14a, were misidentified by the EMSD algorithm. The possible reasons for this are as follows. First, as shown in Figure 14b, the difference between the misidentified parts was very small, and the recognition accuracy was limited by the resolution of the camera used. Second, sheet metals may additionally undergo slight stress or deformations during manufacturing and transportation, further increasing the difficulty of distinguishing between similar parts. However, as shown in Figure 14c, on the photo taken at another aspect angle, the results of the F-R and F-L parts using the EMSD algorithm were correct. As indicated by the matching result plot, the algorithm described the differences between the pair of parts very well. This shows that in practical applications, the algorithm could provide higher recognition accuracy if there was suitable vision configuration and with enough angle aspects. We confidence that, with the proposed EMSD algorithm, multiple image groups from multi-eye cameras should greatly improve the recognition accuracy and achieve better robustness.

## 6. Conclusions

This study entailed the development of the effective mean square differences (EMSD) algorithm and demonstrated its effectiveness in matching highly similar sheet metal parts. With its faster matching speed and great accuracy, this algorithm has remarkable potential in practical applications in sheet metal production and other engineering fields, representing an important step towards accurate and efficient matching of highly similar parts. However, there are still challenges to be addressed in improving matching accuracy, especially in the presence of interference between similar and symmetrical parts. Future research should focus on several aspects. First, more advanced deep learning techniques can be incorporated to enhance the robustness and accuracy of image matching, especially in the presence of distractors and complex backgrounds. Furthermore, more extensive experiments and comparisons with other state-of-the-art methods can provide valuable insights into the strengths and limitations of the algorithm. Further research can continue to advance the field of similar part recognition and pave the way for more advanced applications in industrial applications and beyond.

## Figures and Tables

**Table 1:** Introduction of test image types.

Test Image	Processing Type
Category 1	Original simulation image
Category 2	Salt-and-pepper noise (20,000, 40,000)
Category 3	Gaussian blur (3 × 3, 5 × 5)
Category 4	Rotate the image in the range of 0°–180°, with a rotation step of 10°
Category 5	Adjust image contrast with contrast gain factors (0.8, 1.3)

**Table 2:** Effective matching scores.

	Effective Matching Score Values									
	A-R	A-L	B-R	B-L	C-R	C-L	D-R	D-L	E-R	E-L
Original Effective Match Score	0.247	0.257	0.276	0.286	0.286	0.257	0.258	0.296	0.286	0.284
Symmetrical Part Effective Match Score	0.257	0.286	0.304	0.314	0.315	0.286	0.287	0.324	0.314	0.283

**Table 3:** Influence of effective matching scores on image matching results.

Sheet Metal Parts	Original Match Score	Results	Effective Match Score	Results
A-R	0.401	0.103	0.391	0.112
A-L	0.354	0.091	0.382	0.109
B-R	0.325	0.093	0.317	0.101
B-L	0.325	0.094	0.316	0.101
C-R	0.388	0.111	0.387	0.122
C-L	0.374	0.096	0.372	0.107
D-R	0.405	0.104	0.397	0.114
D-L	0.329	0.094	0.324	0.102
E-R	0.332	0.095	0.323	0.102
E-L	0.370	0.105	0.374	0.106

**Table 4:** Comparison of the recognition accuracy of different algorithms [%].

Algorithm	Original	Salt and Pepper	Gaussian	Contrast	Rotation	Total
VGG-16	70.0	69.2	68.1	70.5	66.7	68.1
**EMSD**	**99.9**	**91.9**	**98.5**	**92.8**	**99.8**	**97.1**
MSD	99.9	77.9	84.6	76.1	92.0	85.6
Sub-NCC	99.9	85.1	87.9	91.2	99.3	93.5
NCC	99.9	80.8	89.4	93.3	91.2	89.5
SSDA	99.9	64.4	70.2	87.1	91.7	82.5

## Data Availability

Data that support the findings of this study are available from the corresponding author upon reasonable request.

## References

[1] Guan J., Xie J. (2018). Analysis of post-processing technology for aviation sheet metal parts. Aeronaut. Precis. Manuf. Technol..

[2] Wang Y., Li Z.Q., Yao J. (2021). Research and application of forming technology for deep-cavity aerospace sheet metal parts. Manuf. Technol. Mach. Tool..

[3] Zhu J.H., Qu P.L., Gong J. (2015). Processing technology for aerospace saddle-shaped sheet metal parts. Mach. Met. Form..

[4] Liu M.Y., Tuzel O., Veeraraghavan A., Taguchi Y., Marks T.K., Chellappa R. (2012). Fast object localization and pose estimation in heavy clutter for robotic bin picking. Int. J. Robot. Res..

[5] Zhu W., Zhang H., Eastwood J., Qi X., Jia J., Cao Y. (2023). Concrete crack detection using lightweight attention feature fusion single shot multibox detector. Knowl. Based Syst..

[6] Billiot B., Cointault F., Journaux L., Simon J.C., Gouton P.J. (2013). 3D image acquisition system based on shape from focus technique. Sensors.

[7] Chang R.S., Chiu J.H., Chen F.P., Chen J.C., Yang J.L. (2011). A Parkinson’s disease measurement system using laser lines and a CMOS image sensor. Sensors.

[8] Stryczek R. (2020). Finite Point Sets in Recognizing Location and Orientation of Machine Parts of Complex Shapes.

[9] Li B. (2018). Research on geometric dimension measurement system of shaft parts based on machine vision. J. Image Video Proc..

[10] Zheng Z.B., Ling Y., Xiao J. (2023). Real-time Recognition Method for Automobile Production Line Parts based on Binocular Vision. Mach. Build. Autom..

[11] Liu C.F., Wang J.B. (2016). Current situation and key technical solutions for informationization of aircraft sheet metal forming. Aeronaut. Manuf. Technol..

[12] Zhang H., Luo R., Luo L., Li K., Fang X., Zhang S. (2023). Deep Learning for Drawing Numbering in Engineering Drawing Management: A Case Study for Refrigerated Compartment Product. Int. J. Patt. Recogn. Artif. Intell..

[13] Kajal G., Tyagi M.R., Kumar G. (2023). A review on the effect of residual stresses in incremental sheet metal forming used in automotive and medical sectors. Mater. Today Proc..

[14] Lü Z.Y., Deng T., Zhang J. (2020). Cross-granularity recognition method for aircraft sheet metal parts based on machine vision. Chin. J. Sci. Instrum..

[15] Cusano C., Napoletano P. (2017). Visual recognition of aircraft mechanical parts for smart maintenance. Comput. Ind..

[16] Guo F., Jin W.Y., Wang M. (2019). Image recognition of mechanical parts based on the improved Faster R-CNN algorithm. J. Mach. Des..

[17] Wei H., Bo W. A template matching algorithm for high precision positioning. Proceedings of the Positioning 8th IEEE International Conference on Software Engineering and Service Science (ICSESS).

[18] Eastwood J., Newton L., Leach R., Piano S. (2022). Generation and categorisation of surface texture data using a modified progressively growing adversarial network. Precis. Eng..

[19] Liu X., Wang Z., Wang L., Huang C., Luo X.J.E. (2021). A hybrid rao-NM algorithm for image template matching. Entropy.

[20] Wang Y., Wang L., Zhang H., Gu Y., Ye Y.J.M. (2022). A novel algorithm for thickness prediction in incremental sheet metal forming. Materials.

[21] Dijitc B. (1972). A class of algorithms for fast digital image registration. IEEE Trans. Comput..

[22] Atallah M.J. (2001). Faster image template matching in the sum of the absolute value of differences measure. IEEE Trans. Image Process..

[23] Wu P., Li W., Fast S.W. (2019). Fast, accurate normalized cross-correlation image matching. J. Intell. Fuzzy Syst..

[24] Zhang Y., Zhang Z., Peng S., Li D., Xiao H., Tang C., Miao R., Peng L. (2022). A rotation invariant template matching algorithm based on Sub-NCC. Math. Biosci. Eng..

[25] Li Z.X., Zhang Z.B., Zhang X., Li B., Huang W.B. (2020). Recognition of sheet metal features and process scheduling based on forming process. J. Netshape Form. Eng..

[26] Adelson E.H., Anderson C.H., Bergen J.R., Burt P.J., Ogden J.M.J. (1983). Pyramid methods in image processing. RCA Eng..

[27] Sun L.J., Fan J. (2018). Target detection of fiber optic transceiver PCB board based on template matching. Comput. Appl. Softw..

[28] He Y.P., Liu X., Cai Y., Yu Z. Research on aided navigation based on terrain elevation matching and simulation. Proceedings of the Chinese Society for Optical Engineering Conferences.

[29] Zhang X.J. (2016). Research on Underwater Robot Geomagnetic Assisted Navigation Algorithm.

[30] Forsyth D. (2014). Object Detection with Discriminatively Trained Part-Based Models. Computer.

[31] Hu J.T., Huang F., Zhang C., Liu B.Q., Wang J. (2014). Influence of down-sampling on super-resolution reconstruction effect of compound eye image. Laser J..

[32] Blu T., Thévenaz P., Unser M. (2004). Linear interpolation revitalized. IEEE Trans. Image Process..

[33] Lehmann T.M., Gönner C., Spitzer K.J. (2001). Addendum: B-spline interpolation in medical image processing. IEEE Trans. Med. Imaging.

[34] Nayak R., Patra D. Image interpolation using adaptive P-spline. Proceedings of the 2015 Annual IEEE India Conference (INDICON).

[35] Zhang H., Eastwood J., Isa M., Sims-Waterhouse D., Leach R., Piano S. (2021). Optimisation of camera positions for optical coordinate measurement based on visible point analysis. Precis. Eng..

[36] Shimin X., Libang Q., Wenyao L., Libin W., Zuyang Z. (2008). MAGCOM and simulation of basic matching algorithm. Aerosp. Control..

